# Farrerol Induces Cancer Cell Death via ERK Activation in SKOV3 Cells and Attenuates TNF-α-Mediated Lipolysis

**DOI:** 10.3390/ijms22179400

**Published:** 2021-08-30

**Authors:** Jongbeom Chae, Jin Soo Kim, seok tae Choi, Seul Gi Lee, Oyindamola Vivian Ojulari, Young Jin Kang, Taeg Kyu Kwon, Ju-Ock Nam

**Affiliations:** 1 Department of Food Science and Biotechnology, Kyungpook National University, Daegu 41566, Korea; chejongbum@naver.com (J.C.); hoyeendahmolar@gmail.com (O.V.O.); 2 National Development Institute of Korean Medicine, 94, Gyeongju-si 38540, Korea; 1211pepero@naver.com; 3 Department of Pharmacology, College of Medicine, Yeungnam University, Daegu 42415, Korea; tjrxopk@naver.com (s.t.C.); yjkang@med.yu.ac.kr (Y.J.K.); 4 Department of Immunology, School of Medicine, Keimyung University, Daegu 42601, Korea; lsg100479@naver.com (S.G.L.); kwontk@dsmc.or.kr (T.K.K.); 5 Center for Forensic Pharmaceutical Science, Keimyung University, Daegu 42601, Korea; 6 Institute of Agricultural Science & Technology, Kyungpook National University, Daegu 41566, Korea

**Keywords:** antitumor effect, adipogenesis, cachexia, cell cycle arrest, farrerol, ovarian cancer

## Abstract

Farrerol (FA) is a flavanone isolated from the Chinese herbal medicine “Man-shan-hong” (*Rhododendron dauricum* L.). In the present study, FA decreased the viability of SKOV3 cells in a dose- and time-dependent manner, and it induced G2/M cell cycle arrest and cell apoptosis. Cell cycle distribution analysis via flow cytometry showed that FA decreased G1 populations and increased G2/M populations in SKOV3 cells. Additionally, Western blotting confirmed an increase in the expression level of proteins involved in the cell cycle, e.g., CDK and cyclins. FA-induced apoptosis in SKOV3 cells was also investigated using a TUNEL assay, and increased expression levels of proapoptotic factors, including Caspase-3 and poly ADP ribose polymerase (PARP), through the Extracellular signal-regulated kinase (ERK)/MAPK pathway were investigated. Proinflammatory cytokines (e.g., IL-6, TNF-α, and IL-1) have been identified as a driver of the pathological mechanisms underlying involuntary weight loss and impaired physical function, i.e., cachexia, during cancer; in the present study, we showed that farrerol attenuates TNF-α-induced lipolysis and increases adipogenic differentiation in 3T3-L1 cells. Thus, farrerol could potentially be used as an anticancer agent or anticachetic drug.

## 1. Introduction

One of the most common and fatal gynecologic malignancies in women is ovarian cancer, with the most common type being epithelial ovarian cancer [1]. Approximately 70% of epithelial ovarian cancers are diagnosed at the terminal stage because effective early detection is difficult; moreover, they are characterized by rapid progression and recurrence [2]. In addition, resistance to drug treatments has increased the lethality of gynecologic malignant tumors. Although the diagnosis and treatment of ovarian cancer has improved significantly over the past 30 years, the 5-year survival rate of women diagnosed with ovarian cancer remains at <50%. Currently, treatment prolongs survival, but ovarian cancer nevertheless has a high probability of recurrence and progression. Therefore, it is necessary to develop new chemotherapy regimens for ovarian cancer [3].

The major regulatory mechanism of cell proliferation is cell cycle control; through the inhibition of cancer cell growth, cell cycle control is one of the most effective methods of cancer treatment [4]. Several cytotoxic and/or DNA-damaging agents that arrest the cell cycle in the G0/G1, S, or G2/M phases and consequently induce apoptosis of cancer cells have been identified. Of these agents, the Cyclin/CDK families have been found to play important roles in G2 to M phase progression, particularly at the end of the G2 phase, when a threshold level of the active CyclinB1/CDK1 complex is reached after the induction of cell apoptosis [5]. Apoptosis is an essential regulatory factor in tissue homeostasis that causes selective cell loss. Apoptotic cells are characterized by morphological and biochemical changes of characteristic features such as DNA fragmentation, apoptotic bodies, and cell shrinkage. Cysteine aspartic acid proteases (caspases) are the major regulators of apoptosis. They are synthesized as an inactive form of the proenzyme in the intracellular environment and are then activated by self-cleavage or cleavage by other caspases. Cleaved Caspase-3 acts as an effector caspase that induces apoptosis by degrading proteins such as DNA fragmentation factor and poly ADP ribose polymerase (PARP) that is found in the nucleus and plays an important role in maintaining the survival of cells involved in DNA repair. PARP is a major target protein of Caspase-3 and is inactivated when cleaved by Caspase-3, promoting cell degradation and causing apoptosis [6,7,8,9,10]. Extracellular signal-regulated kinase (ERK), c-Jun N-terminal kinase (JNK), and p38 MAPK function in protein kinase cascades and play critical roles in the regulation of cell proliferation, differentiation, and apoptosis [11]. The ERK signaling pathway can be activated in response to various extracellular stimuli such as growth factors, mitogens, and cytokines, and it is an important target in the diagnosis and treatment of cancer [12]. JNK is potently and preferentially activated by a variety of environmental stressors including UV, ceramides, and inflammatory cytokines [13].

In cancer patients, cancer cachexia is a wasting syndrome that can include anorexia, fatigue, metabolic and endocrine alterations, and loss of lean body mass. This syndrome decreases the efficacy of conventional chemotherapy and radiotherapy, reduces cancer patient quality of life, and worsens their prognosis. Indeed, at least 20% of cancer patients die from cachectic symptoms such as weight loss. Therefore, to overcome cancer, it is necessary to suppress cancer cells directly and control cancer-induced cachexia, particularly for the preservation of lean body mass [14]. In recent years, the pathophysiological mechanisms underlying adipose tissue wasting during cancer cachexia have been intensely investigated; consequently, the key processes and therapeutic targets have been identified. These include proinflammatory cytokines secreted from the tumor cell themselves and from the host cells in response to tumors. Although many studies have been conducted with the aim of developing treatments for cachexia, approved drugs do not currently exist. Likewise, patients with ovarian cancer was reported about cachexia including muscle depletion, malnutrition and abnormal lipid metabolism. Most studies of cachexia caused by ovarian cancer were about changing muscles, however, there have been no studies on changing lipid metabolism [15,16,17]. As a master regulator of adipocyte differentiation, peroxisome proliferator-activated receptor (PPAR) γ regulates adipogenic genes during differentiation and controls lipid metabolism. PPAR γ agonists, especially thiazolidinedione derivatives, e.g., rosiglitazone, pioglitazone, and troglitazone, increase the number of adipocytes that produce adiponectin while decreasing the number of adipocytes that express TNF-α, IL-6, and other adipokines, which negatively regulate insulin sensitivity. Additionally, these PPAR γ agonists have been reported to attenuate cancer-induced body weight wasting and TNF-α-induced adipocyte wasting. Rosiglitazone has been approved for the treatment of type 2 diabetes by the Food and Drug Administration (FDA); however, because rosiglitazone can cause or exacerbate congestive heart failure as well as several other serious side effects, the FDA has restricted its use. Although pioglitazone is currently available for clinical use, it is associated with safety concerns. Thus, safe agents that target PPAR γ activation are required for the prevention and treatment of cachexia [18,19,20,21,22]. In previous reports, structure–activity relationship data revealed that several chalcones, i.e., biosynthetic precursors of flavonoids derivatives, exhibit higher activity than rosiglitazone, which suggests the possibility that chalcone derivatives could be developed as PPAR γ agonists [23]. However, adipocyte regulation and improved lipid wasting through PPAR γ agonists of other flavonoids have yet to be reported. Farrerol (FA) is a flavanone purified from the Chinese herbal medicine “Man-shan-hong” (*Rhododendron dauricum* L.) [24]; it has been reported to exert biological effects including antioxidative, anti-inflammatory, antibacterial, and anti-angiogenesis, as well as the inhibition of vascular smooth muscle cell proliferation, amelioration of type 2 diabetes, and anti-cancer effects in the SGC-7901 human gastric cancer cell line [9,10,25,26,27,28]. In the present study, we investigated the potential anticancer effect of FA in SKOV3 cells as well as the regulatory effects of adipogenesis and TNF-α-induced lipid wasting.

## 2. Materials and Methods

### 2.1. Reagents and Antibodies

FA (CAS Number: 24211-30-1, Figure 1) was purchased from Chemfaces (Wuhan, China). Roswell Park Memorial Institute medium-1640 (RPMI-1640), Dulbecco’s modified Eagle’s medium-high glucose (DMEM-H), and fetal bovine serum (FBS) were obtained from Gibco Life Technologies (Grand Island, NY, USA). Moreover, 3-[4,5-Dimethylthiazol-2-yl]-2,5-diphenyltetrazolium bromide (MTT) was purchased from Amresco (Solon, OH, USA). Antibodies against CDK1, CDK2, CDK4, Cyclin A, Cyclin B, Cyclin D, Cyclin E, Caspase-3, PARP, p38, p-p38, ERK, p-ERK, JNK, p-JNK, PGC1α, UCP-1, PPARγ, and β-actin were purchased from Cell Signaling Technology (Beverly, MA, USA) and Santa Cruz Biotechnology (Santa Cruz, CA, USA).

### 2.2. Cell Culture and Treatment

Human ovarian epithelial cancer SKOV3 cells and human endothelial EA.hy926 cells were obtained from the Korea Cell Line Bank (Seoul, Korea). SKOV3 cells were grown in RPMI-1640 supplemented with 10% FBS. EA.hy926 cells were grown in DMEM-H supplemented with 10% FBS. All cells were cultured under a humidified atmosphere with 5% CO_2_ at 37 °C. SKOV3 and EA.hy926 cells were treated over different time periods with a concentration gradient of FA (40–160 μM) dissolved in medium with 10% FBS.

### 2.3. Crystal Violet Staining Assay

Crystal violet staining assay was used to evaluate whether FA affects cell proliferation. Briefly, cells were seeded in 6 well plates with RPMI medium conteined 10% FBS at a density of 2 × 105 cells per well, then incubated with a concentration gradient of FA (40–160 μM) or DMSO as vehicle for 24 or 48 h. Cells were then washed once with PBS, after which they were fixed with 4% paraformaldehyde for 15 min and stained with saturated crystal violet solution for 20 min at room temperature. After that, crystal violet solution was removed and cells were washed twice with tap water, then samples were photographed using a camera in optical microscope.

### 2.4. Cell Viability (MTT) Assay

SKOV3 cells were seeded at a density of cells per well in 96-well plates and maintained at 37 °C in a CO_2_ incubator. After incubation for 24 h, the cells were treated with 0, 40, 80 and 160 μM of FA. The control cells were treated with 4 μL/mL of DMSO alone in medium. After incubation for 24 or 48 h, the medium was removed and 0.5 mg/mL of MTT solution in phosphate-buffered saline (PBS) was added to each well. After incubation for 3 h, the MTT solution was removed and Propan-2-ol was added to each well. After incubation for 1 h, the absorbance at 595 nm was measured using an ELISA reader (Infinite f50, TECAN, Männedorf, Switzerland).

### 2.5. Cell Cycle Analysis by Flow Chemistry

Cell cycle distribution was analyzed by flow cytometry (FACS) after staining with propidium iodide (PI) solution (BD Pharmingen™). SKOV3 cells (2 × 10^5^ cells) were seeded into a 60-mm culture dish and treated with each concentration of FA (control, 40, 80, and 160 μM) for 24 or 48 h. Subsequently, the cells were harvested, washed in cold PBS, and fixed with 70% ethanol at 4 °C overnight. The fixed cells were exposed to RNase A and PI for 30 min, and then the cell cycle was investigated using flow cytometry (BD FACS Canto II).

### 2.6. Annexin V-FITC/PI Staining Assay

The SKOV3 cells were seeded into a 100-mm culture dish (2×10^5^ cells/mL). After cell adherence, they were treated with FA (0, 40, 80, and 160 μM) for 24 or 48 h. Then, the cells were collected and prepared into the suspension. Annexin V-FITC and Propidium Iodide(PI) (Annexin V-FITC Apoptosis Detection Kit II, BD 556570, BD science) were added and mixed. After staining, light was avoided for 5–15 min at room temperature, and then apoptotic cells were detected by flow cytometry (Attune NxT; ThermoFisher, Eugene, OR, USA).

### 2.7. TUNEL Assay

SKOV3 cells were seeded onto a chamber slide (Lab-Tak II chamber slider system, Nalge Nunc Int., Naperville, IL, USA) at a density of 2 × 10^4^ cells/well. The cells were treated with FA at 0, 40, 80, or 160 µM. After incubation for 24 or 48 h, the cells were fixed by using 6% glutaraldehyde for 10 min and 70% EtOH for 10 min. A permeabilization step was then completed using 0.25% Triton X-100 in PBS for 15 min. TUNEL staining was performed according to the product manual using an in situ BrdU-Red DNA Fragmentation Kit (Abcam, ab66110, Cambridge, MA, USA). DAPI staining was then performed with a mounting solution containing 1-µg/mL DAPI and photographed using a fluorescence microscope.

### 2.8. Western Blot Analysis

Cells were lysed in lysis buffer (RIPA buffer) containing protease inhibitor and phosphatase inhibitor cocktails. Equal amounts of protein were separated on 10% or 15% sodium dodecyl sulfate polyacrylamide gels. Proteins were transferred to nitrocellulose membranes, blocked in 5% skim milk in TBST (10-mM Tris at pH 8.0, 150-mM NaCl, and 0.05% Tween 20) for 1 h, and incubated overnight with each primary antibody at 4 °C. After washing with TBST, the membranes were incubated with horseradish peroxidase-conjugated secondary antibodies at room temperature for 1 h. Proteins were detected using an enhanced chemiluminescence reagent (GE Healthcare, Buckinghamshire, UK). ImageJ software (http://imagej.nih.gov/ij/) was used to quantify the reported protein expressions. All experiments were performed in triplicate. Expression was considered to be significant at *p* < 0.05 according to one-way ANOVA.

### 2.9. Colorimetric Caspase-3 Activity Assay

Colorimetric Caspase-3 assay kits were obtained from Abcam (cat. no. ab39401) and used to measure the caspase activity following the manufacturer’s instructions. Briefly, 1 × 10^6^ SKOV3 cells/mL were incubated at 37 °C for 30 min with 50 μM of each MAPK inhibitor: PD98059 (MEK1, ERK inhibitor), SB20380 (p38 inhibitor), and SP600925 (JNK inhibitor). After pretreatment with the inhibitors, the culture medium was changed to fresh medium containing FA (160 μM) with or without each MAPK inhibitor. After washing with cold PBS, cold cell lysis buffer was used to lyse cells for 15 min and then the supernatant was separated by centrifugation (12,000× *g* at 4 °C for 10 min). The cell lysate was added to assay plates containing reaction buffer with 10 µL of acetyl-Asp-Glu-Val-Asp *p*-nitroanilide as a substrate for Caspase-3, before being incubated at 37 °C in the dark for 1.5 h. Finally, it was measured with a microplate reader at 405 nm to quantify the formation of *p*-nitroanilide, and the relative increases of Caspase-3 activity were calculated through comparison with the control group.

### 2.10. Adipocyte Differentiation and Treatments

Differentiation into mature adipocytes was initiated by culturing 3T3-L1 preadipocytes for 2 days after reaching 100% confluence. It was induced by changing the culture medium to DMEM supplemented with 0.5-mM 3-isobutyl-1-methylxanthine, 0.25-µM dexamethasone, 167-nM insulin, 100-µM indomethacin, and 10% FBS for 2 days. After 2 days, the medium was changed to DMEM containing 10% FBS and 10-µg/mL insulin for 8 days, with medium changes conducted every 2 days. During the differentiation process, 3T3-L1 cells were treated every 2 days with FA at concentrations of 15 or 30 µg/mL.

### 2.11. Oil Red O Staining

Cells were washed with PBS and fixed with 5% formalin for 1 h at room temperature. They were then washed with 60% Propan-2-ol, stained with 0.4% ORO for 10 min at room temperature, washed 4 times with distilled water, and photographed with a microscope camera at 100× magnification. Images of three random fields from three replicate wells were obtained. Cells stained with Oil Red O solution were dissolved in Propan-2-ol and their absorbance was measured at 495 nm.

### 2.12. Triglyceride Assay

Triglyceride content was assessed with a colorimetric/fluorometric assay kit (Biovision, Milpitas, CA, USA) according to the manufacturer’s instructions. Briefly, 3T3-L1 preadipocytes were seeded into 6-well plates with differentiation media and FA (15 or 30 µM) for up to 8 days. After differentiation, cells were lysed in 5% NP-40 lysis buffer, and triglycerides were converted to fatty acids and glycerol with a lipase enzyme. The absorbance of the released glycerol was measured at 570 nm.

### 2.13. Real-time Reverse Transcription Polymerase Chain Reaction (RT-PCR)

After differentiation of 3T3-L1 cells with FA (15 or 30 µM), total RNA was extracted from adipocytes using Trizol reagent. A cDNA library was synthesized with a PrimeScript™ RT Reagent Kit (TakaRa, Shiga, Japan). Subsequently, mRNA expression levels were quantified by analysis of cDNA implemented with an iCycler iQ™ Real-Time PCR Detection System (Bio-Rad Laboratories, Hercules, CA, USA) using SYBR Green (TOYOBO, Osaka, Japan). The conditions of the PCR reaction were as follows: a denaturation cycle at 95 °C for 10 min, followed by 95 °C for 15 s and 60 °C for 10 min. mRNA expression levels were normalized to β-actin and relative gene expression was expressed as fold change in mRNA expression levels. The sequences of primers used in the experiment are shown in Table 1.

### 2.14. Statistical Analysis

All statistical analyses were conducted in SPSS version 25.0 (SPSS Inc., Chicago, IL, USA). The differences between groups were analyzed using one-way ANOVA and *p* values < 0.05 were considered statistically significant.

## 3. Results

### 3.1. Inhibitory Effects of FA on Cell Proliferation via G2/M Cell Cycle Arrest in SKOV3 Cells

**Figure 2:**
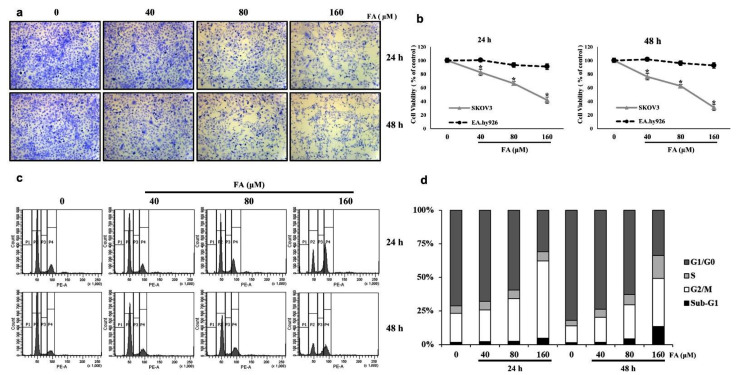
Farrerol (FA) inhibits cell proliferation of SKOV3 cells through G2/M cell cycle arrest. (**a**) Crystal violet-stained cell images of SKOV3 cells after treatment with the indicated concentration of FA for 24 and 48 h. (**b**) The cell viability of SKOV3 and EA.hy926 on FA treatment cells was measured via an MTT assay. Data are means ± standard deviation from three independent experiments (*, *p* < 0.05). (**c**) SKOV3 cells were treated with FA (0, 40, 80, or 160 μM) for 24 or 48 h, harvested, and digested with RNase. Cellular DNA was stained with propidium iodide, and the cell cycle distribution was analyzed using flow cytometry. (**d**) Cell cycle distribution data.

To investigate the effect of FA on SKOV3 cell proliferation, cells were treated with 0, 40, 80, and 160 μM of FA for 24 and 48 h. Then, the cells were stained to crystal violet dye and observed under an optical micro-scope (×100 magnification). The confluence of the treated SKOV3 cells was lower than that of the control cells (Figure 2a). Additionally, an MTT assay indicated that the FA treatment decreased the viability of SKOV3 cells in a dose- and time-dependent manner. However, the normal control cells, i.e., human endothelial EA.hy926 cells, did not show any significant change in cell viability after treatment with FA (Figure 2b). According to a flow cytometric analysis, treatment of SKOV3 cells with FA for 24 h caused the percentage of cells in the G0/G1 phase to decrease and the percentage in the G2/M phase to increase, each in a dose-dependent manner (Figure 2c). At 0-, 40-, 80-, and 160-μM FA, the percentage of cells in the G0/G1 phase was 59.6%, 51.7%, 44.6%, and 21.5%, respectively, whereas the percentage of cell populations in the G2/M phase was 17.9%, 17.7%, 23.8%, and 39.9%, respectively. These results indicate that FA reduces G0/G1-phase populations in SKOV3 cells and induces G2/M phase arrest, which suggests a possible mechanism by which the inhibitory effect of FA on cancer cell growth occurs. However, after treatment of SKOV3 cells with 160-μM FA for 48 h, the population of Sub-G1 phases but not G2/M phases tended to increase. This shows that treatment with 160-μM FA for 48 h induces apoptosis of SKOV3 cells rather than cell cycle arrest (Figure 2d).

### 3.2. Effects of FA on the Expression of Cell Cycle Regulatory Proteins in SKOV3 Cells

**Figure 3:**
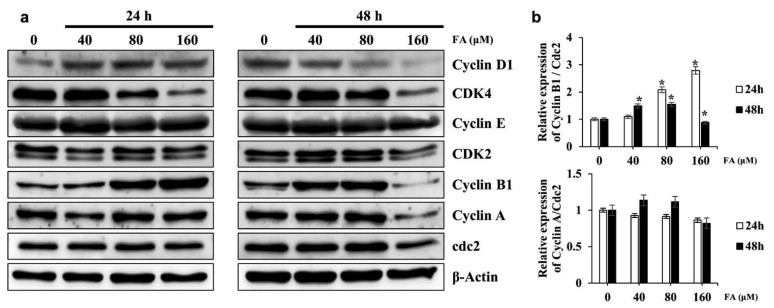
Effect of farrerol (FA) on cell cycle regulatory proteins in SKOV3 cells. (**a**) SKOV3 cells were treated with FA (0, 40, 80, or 160 μM) for 24 or 48 h and then harvested. The cell lysates were prepared, subjected to SDS-PAGE, and analyzed by Western blotting. The expression of Cyclin D1, CDK4, Cyclin E1, CDK2, Cyclin B1, Cyclin A, and Cdc2 was determined; the expression of β-actin was used to verify equal loading of the samples. (**b**) Representative image of the Western blots. Data are means ± standard deviation from three independent experiments (*, *p* < 0.05).

According to the Western blot analysis, the expression of Cyclin B1 increased in a dose-dependent manner in the 24-h FA treatment group, but expression decreased with FA treatment at 160 μM for 48 h. The Cyclin A/CDK1 expression ratio did not differ significantly in the FA-treated group. CDK4 expression, involved in the G0/G1 phase, decreased as the dose of FA increased, whereas Cyclin E and CDK2, involved in the S phase, showed no significant changes in terms of expression and ratio (Figure 3a,b). These results support the cell cycle analysis results. However, in SKOV3 cells treated with FA at 160 μM for 48 h, the expression of CDK, Cyclin B1, and Cyclin A markedly decreased; therefore, FA treatment at 160 μM for 48 h seems to be more effective for apoptosis than for cell cycle arrest.

### 3.3. FA Induces Apoptosis in SKOV3 Cells

**Figure 4:**
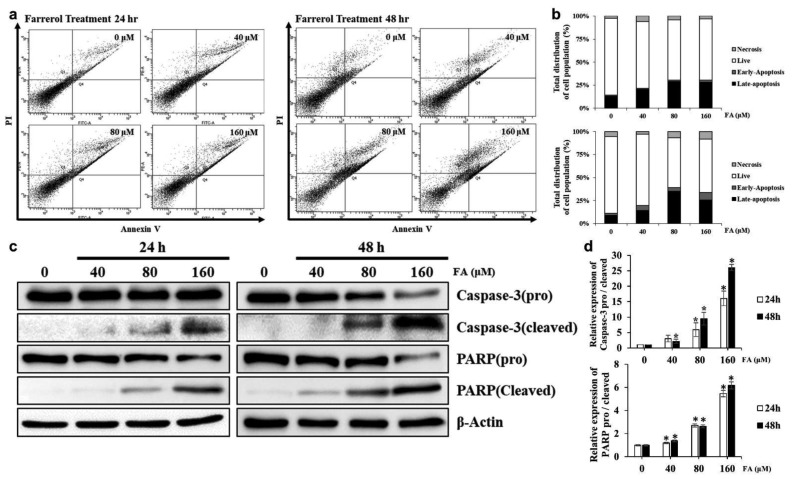
Effect of farrerol (FA) on the induction of apoptosis in SKOV3 cells. (**a**) SKOV3 cells were treated with the indicated concentration of FA for 24 or 48 h. Apoptotic cells were detected using a Annexin V-FITC/PI staining assay. (**b**) Quantification of apoptotic cell ratio of Annexin V/PI staining assay. (**c**) SKOV3 cells were treated with FA for 24 or 48 h. The cells were lysed and total proteins were separated by SDS-PAGE. Equal loading was confirmed by the quantification of β-actin. (**d**) Representative images of western blots. Data are means ± SD from three independent experiments (*, *p* < 0.05).

To determine the effect of inducing apoptosis by FA treatment on SKOV3 cells, were stained to Annexin V and PI. After staining apoptotic cells were detected by flow cytometry. The apoptotic cells appeared fluorescence due to phosphatidylserine (PS) ectropion on the cell membrane and were combined with FITC-labeled Annexin V. The apoptotic cells in the late stage and dead cells have an incomplete cell membrane structure, and thus PI was combined with the cell nucleus through the cell membrane. As a result, the combination of Annexin V-FITC and PI could differentiate the cells in the stages of apoptosis. Result showed a significant elevation in the number of apoptotic cells by FA treatment (Figure 4a,b).

Additionally, DNA fragmentation visualized by TUNEL and DAPI staining, was detected in apoptotic cells. TUNEL-positive cells also increased dose- and time-dependently in SKOV3 cells treated with 0, 40, 80, and 160 μM of FA for 24 or 48 h (Figure S1).

Protein expression of cleaved Caspase-3, an activated form of Caspase-3, was examined by Western blotting to identify the molecular mechanism by which FA induced apoptosis in SKOV3 cells. The expression of cleaved aspase-3 significantly increased in SKOV3 cells after FA treatment in a dose- and time-dependent manner. As the expression of cleaved Caspase-3 increased, the expression of cleaved PARP, the inactive form of PARP, also increased with increasing FA concentrations. As the activity of Caspase-3 was increased by FA, the expression of cleaved PARP, the active form of the target protein PARP, also significantly increased with increasing FA concentrations (Figure 4c,d).

### 3.4. Apoptosis by FA Treatment Mediates ERK MAPK Signaling in SKOV3 Cells

**Figure 5:**
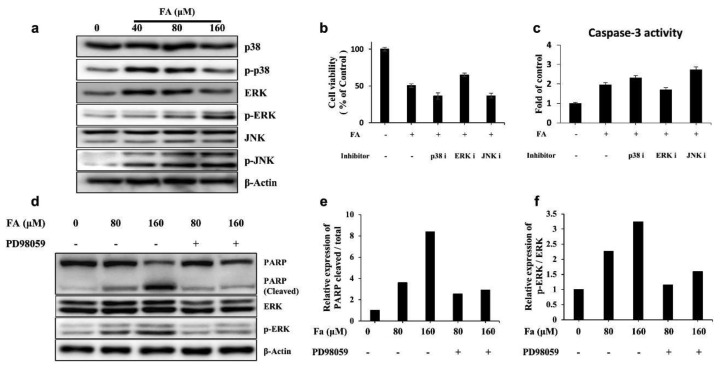
Apoptotic effect of farrerol (FA) in SKOV3 was mediated by the ERK MAPK pathway. (**a**) Expression levels of MAPK in FA-treated SKOV3 cells were detected using Western blotting. (**b**) The viability of SKOV3 cells following treatment with each MAPK inhibitor with or without FA (160 μM) was measured via an MTT assay. (**c**) Caspase-3 activity of SKOV3 cells following treatment with each MAPK inhibitor with or without FA (160 μM), as measured by a colorimetric Caspase-3 activity assay. (**d**) Expression levels of PARP, cleaved PARP, ERK, and phospho-ERK in FA-treated SKOV3 cells with or without PD98059, as detected using Western blotting. (**e**) Expression levels of the cleaved PARP/PARP ratio. (**f**) Expression levels of the p-ERK/ERK ratio. All data are means ± standard deviation from three independent experiments.

Apoptosis is reportedly regulated by the MAPK signaling pathway [29]. Thus, to examined whether this pathway accounts for FA-induced apoptosis induction. The Western blot results (Figure 5a) showed that FA treatment upregulated phosphorylated ERK and JNK in a dose-dependent manner in SKOV3 cells. However, the phosphorylation level of p38 MAPK was decreased by FA treatment. To determine the MAPK pathway that regulated induction of apoptosis by FA treatment, the cell viability and Caspase-3 activity were measured by treatment with each inhibitor: PD98059 (MEK1, ERK inhibitor), SB20380 (p38 inhibitor), and SP600125 (JNK inhibitor). The cotreatment of ERK with FA inhibitors alleviated cell death and downregulated Caspase-3 activity in SKOV3 cells. However, p38 and JNK inhibitor did not affect cell viability and Caspase-3 activity (Figure 5b,c). This result suggests that FA-induced apoptosis was attenuated by ERK inhibition. As shown in Figure 5d, FA treatment relieved the cleavage levels of PARP in PD98059 cotreatment groups when compared with FA-only treatment groups in SKOV3 cells. In addition, FA-induced phosphorylation of ERK was decreased significantly after cotreatment with PD98059 (Figure 5d–f). These results collectively show that FA-induced apoptosis occurs through activated ERK signaling in SKOV3.

### 3.5. FA Reverses Lipid Wasting by TNF-α in 3T3-L1 Cells

To investigate effect of farrerol on lipid wasting by cancer cachexia, this study measured changing of lipid contents by conditioned medium of SKOV3 (Figure S2). As a result, farrerol could increase lipid contents that SKOV3 CM decreased. Based on this result, it was investigated whether farrerol could improve lipid wasting caused by TNF-*α*, a typical factor of Cachexia, in various cancer types.

TNF-*α* is a negative regulator of adipocytes and well-known factor of cancer cachexia. It reportedly suppresses adipogenic differentiation and upregulates adipocyte browning or lipid wasting. TNF-*α* is upregulated in the adipose tissue of patients with cancer [14] and type 2 diabetes [30]. Thus, it tested whether FA could reverse the inhibitory effects of TNF-*α* on adipocyte differentiation or lipid wasting in well-differentiated adipocytes.

**Figure 6:**
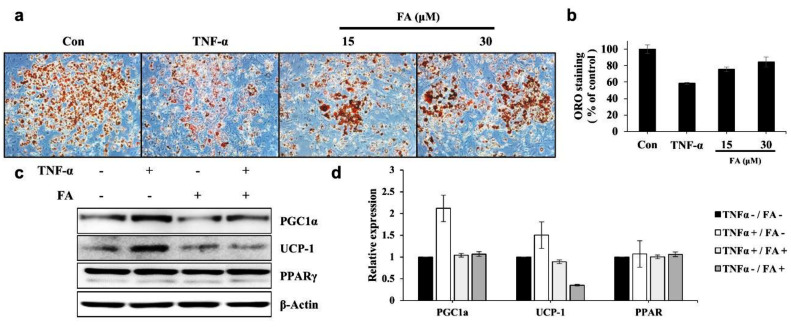
Farrerol (FA) reverses TNF-*α* induced lipid wasting in differentiated 3T3-L1 cells. (**a**) Differentiated 3T3-L1 cells were treated with TNF-*α* (20 ng/mL) with or without FA (15 or 30 µM) for 48 h. The representative images of the Oil Red O (ORO)-stained-cells in each indicated group are shown. (**b**) The ORO staining in the cells was dissolved in Propan-2-ol and measured. (**c**) Expression levels of PGC1a, UCP-1, and PPARγ in differentiated 3T3-L1 cells treated with TNF-*α* with or without FA for 48 h were detected using Western blotting. (**d**) The expression levels of PGC1a, UCP-1, and PPARγ are shown. Equal loading was confirmed by the quantification of β-actin. Data represent the means ± standard deviation from three independent experiments.

As shown in Figure 6a, the incubation of 3T3-L1 adipocytes with TNF-*α* decreased lipid accumulation to approximately 65% of the lipid levels in 3T3-L1 adipocytes treated with fresh culture medium, whereas cotreatment of TNF-*α* with FA decreased lipid accumulation to approximately 80%. This indicates that FA inhibits TNF-*α*-mediated lipolysis (Figure 6a,b). To determine whether FA could regulate TNF-*α*-induced adipocyte browning in 3T3-L1 cells, it was measured expression levels of UCP-1, PGC1α, and PPAR γ by Western blotting. The results showed that only TNF-α increased the expression levels of UCP-1 and PGC1α relative to the untreated group, whereas the FA cotreatment reduced the expression levels of UCP-1 and PGC1α compared with the TNF-α-only treatment groups in SKOV3 cells. The FA-only treatment did not affect the expression levels of UCP-1 and PGC1a (Figure 6c,d). These results show that FA attenuates TNF-*α*-mediated lipid wasting in 3T3-L1 cells.

### 3.6. FA Increases Lipid Accumulation and Adipogenic Differentiation in 3T3-L1 Cells

**Figure 7:**
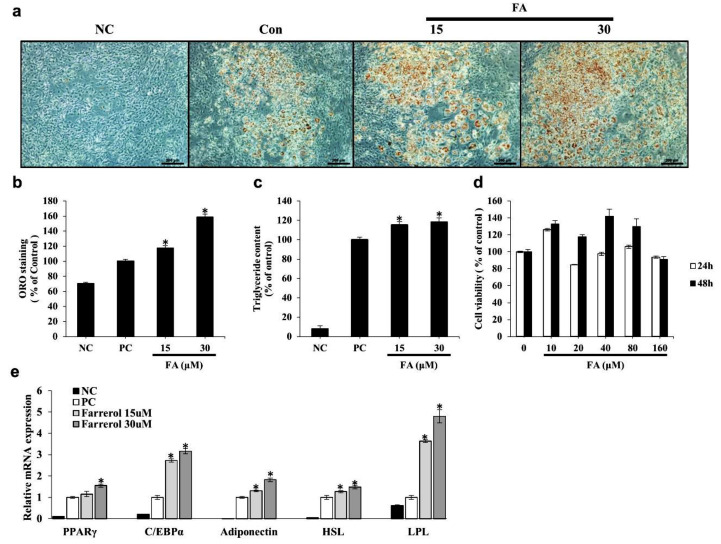
Effects of farrerol (FA) on lipid accumulation, triglyceride content, and adipogenic differentiation in 3T3-L1 cells. (**a**) First, 3T3-L1 preadipocytes were treated with differentiation medium in the presence or absence of FA at 15 or 30 μg/mL for 8 days. Lipid accumulation was quantified by staining with Oil Red O (ORO) solution and absorbance was measured at 495 nm. Differentiated 3T3-L1 cells photographed at 100× magnification after ORO staining. (**b**) Quantification of ORO-stained intracellular lipid content. (**c**) Intracellular triglyceride content quantified by a triglyceride assay kit and assessed at 570 nm. Preadipocytes were used as negative controls (NC), whereas fully differentiated adipocytes were used as positive controls (PC). (**d**) Cell viabilities were determined with an MTT assay. (**e**) The 3T3-L1 preadipocytes were treated with FA at the indicated concentrations for 24 or 48 h. The relative mRNA expression levels of PPARγ, C/EBPα, adiponectin, HSL, and LPL were assessed by real-time PCR. Each experiment was repeated in triplicate. Bars represent means ± standard error (*, *p* < 0.01 compared to control group).

We investigated the effects of FA on adipogenesis by measuring the intracellular lipid accumulation via an ORO-staining assay and an assessment of the triglyceride contents. FA significantly increased adipocyte differentiation and intracellular lipid accumulation in a dose-dependent manner (Figure 7a,b). Furthermore, FA significantly increased intracellular triglyceride content compared with the content measured in control cells (Figure 7c). These results indicate that FA can exert positive effects on adipogenic differentiation. FA treatment during differentiation increased mRNA expression levels of PPARγ, CCAAT/enhancer-binding protein α (C/EBPα), adiponectin, HSL, and LPL in differentiated 3T3-L1 cells (Figure 7e).

## 4. Discussion

This study investigated the effect of FA on SKOV3 cell growth and determined the mechanism of action in relation to its anticancer effects in these human ovarian cancer cells. It was observed that treatment with FA decreased the viability of SKOV3 cells in a time- and dose-dependent manner but confirmed that the viability of noncancerous EA.hy926 cells was not affected by FA. Our results are consistent with a previously reported study by Liu et al., in which FA was found not to be toxic to HUVEC cells [10]. These results show the selective cytotoxicity of FA to cancer cells. In addition, FA-induced cell cycle arrest in SKOV3 cells. In FA-treated SKOV3 cells, the population of G0/G1 cells decreased and the population of the G2/M phase increased in a dose-dependent manner as the concentration of FA increased. However, after a 48-h treatment with FA at 80 or 160 μM, the Sub-G1 population increased but the population of cells in the G2/M phase did not increase.

Using a Western blot analysis, this study found that the proteins involved in cell cycle arrest showed molecular changes that were consistent with our cell cycle analysis results. Changes in Cyclin D1 and CDK4/6, i.e., proteins involved in the G1/G0 phase, included a dose-dependent reduction in CDK4 only after 24 h of FA treatment, but a dose-dependent reduction in Cyclin D1 and CDK4 after 48 h of FA treatment. This shows that the G1/G0 phase population is reduced in a dose-dependent manner by FA exposure. Cyclin E and CDK2, proteins involved in the S phase, were not significantly affected by FA regardless of concentration or time. However, the expression of Cyclin B1 was significantly increased in a dose- and time-dependent manner and then decreased rapidly with a treatment of FA at 160 μM for 48 h. Although, Cyclin A and CDK1 were not significantly affected by FA concentrations or exposure time, their levels rapidly decreased after 48 h of FA treatment at 160 μM.

In previous studies, it was reported that the progression of the cell cycle involves the degradation of Cyclin B and that a subunit of protein kinase CDK1/Cyclin B is required for inactivation of the kinase and exit from mitosis [31]. Cyclin B is degraded by the ubiquitin pathway, a system involved in most selective protein degradation in eukaryotic cells [31,32,33]. In our study, observed an increased level of Cyclin B with FA treatment suggesting that it induced G2/M phase arrest in SKOV3 cells, abrogated the degradation of Cyclin B, and thereby increased Cyclin B levels. However, further studies will be required to confirm this hypothesis.

Figure 3a shown a rapid decrease in the levels of proteins related to the cell cycle when cells were treated with 160-μM FA for 48 h, which is thought to be due to apoptosis. To elucidate the molecular mechanism by which apoptosis occurred in FA-treated SKOV3 cells, the induction of apoptosis by Caspase-3, which is an effector caspase, cleaved Caspase-3, which is an active form, and the PARP cleavage of cleaved Caspase-3 was assessed via Western blotting. The data showed that the expression of cleaved Caspase-3 was significantly increased by FA and that expression of the cleaved form of PARP was also increased [19]. With FA treatment, the activity of Caspase-3 was higher at 24 h than at 48 h, suggesting that FA-induced cell cycle arrest occurred at 24 h and apoptosis was induced over time. In addition, our results suggest that FA-induced apoptosis in SKOV3 cells was mediated via the ERK/MAPK pathway. Cotreatment of ERK with FA inhibitors alleviated cell death and downregulated Caspase-3 activity in SKOV3 cells, whereas p38 and JNK inhibitor did not affect cell viability or Caspase-3 activity. Thus, FA-induced apoptosis seemed to be attenuated by ERK inhibition. In SKOV3 cells, FA treatment also relieved the cleavage levels of PARP in PD98059 cotreatment groups relative to the levels in the group treated with FA only. In addition, FA-induced phosphorylation of ERK was also significantly reduced after cotreatment with PD98059. Overall, these results suggest that FA-induced apoptosis through activation of ERK signaling in SKOV3 cells.

Cancer cachexia, a wasting syndrome (as described earlier; [14]), has various causes including HSP70, HSP90, and PTHrP; among these, proinflammatory cytokines are the most reported cause. In the cancer microenvironment, the proinflammatory and procachectic factors produced by the tumor cells play important roles in the genesis of cachexia. Several proinflammatory cytokines, including IL-1, IL-6, and TNF-α, may have important roles in the pathological mechanisms of cancer cachexia. In the cancer cachexia condition, the carbohydrate, protein, and fat metabolisms are altered. TNF-α is responsible for the increase in gluconeogenesis, adipocyte browning, loss of adipose tissue, and proteolysis, while it also decreases protein, lipid, and glycogen synthesis. It has been associated with the formation of IL-1 and an increase in uncoupling protein-2 (UCP2) and UCP3 expression in skeletal muscle in the cachectic state [34].

PPAR γ agonists, including the thiazolidinedione derivatives rosiglitazone, pioglitazone, and troglitazone, attenuate cancer-induced body weight wasting and TNF-*α*-induced adipocyte wasting [19,20,21,22]. However, these drugs can cause or exacerbate congestive heart failure and lead to several other serious side effects; thus, the FDA has placed restrictions on their use. Therefore, safe agents, such as extracts of natural products or single compounds that target PPAR γ activation, are needed for the prevention and treatment of cachexia. Previously, several chalcones that are biosynthetic precursors of flavonoid derivatives have been suggested as possible PPAR γ agonists, similar to thiazolidinedione derivatives [23]. In addition, the herbal extract SGE has been shown to alleviate skeletal muscle and adipocyte wasting caused by cancer cachexia [35]. Given these findings, it is important to continue identifying and researching materials that could improve skeletal muscle and lipid cachexia, particularly in natural products or single compounds rather than thiazolidinedione drugs. Figure 6 showed that the natural product FA could attenuate TNF-*α* mediated lipolysis, decrease the expression of adipocyte browning-related genes, and increase adipogenic differentiation or lipid accumulation by inducing expression of adipogenesis-related genes. However, further in vivo research will be required on the mechanism of FA as a PPAR γ agonist.

## 5. Conclusions

In conclusion, our results suggest that FA treatment inhibits cell proliferation through cell cycle arrest, induces ERK/MAPK-mediated apoptosis in SKOV3 cells, attenuates TNF-*α* mediated lipolysis, and induces adipogenic differentiation. Further in vivo studies related to cancer and mechanistic studies assessing the role of PPARγ agonists are required; nevertheless, this study suggests that FA could potentially be developed as an anticachetic supplement and an anticancer agent with few known side effects.

## Figures and Tables

**Figure 1:**
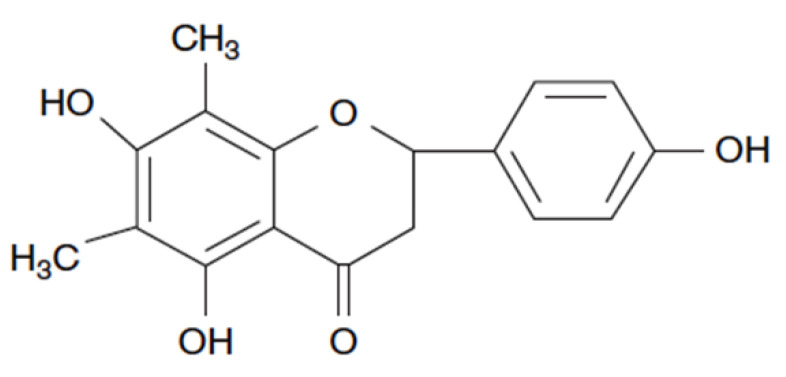
The chemical structure of farrerol.

**Table 1:** Primer sequences for RT-PCR.

Gene Name	Accession No.		Sequence
*PPARγ*	AB644275.1	Forward	5′–GGA AGA CCA CTC GCA TTC CTT–3′
		Reverse	5′–GTA ATC AGC AAC CAT TGG GTC A–3′
*C/EBPα*	BC058161	Forward	5′–CAA GAA CAG CAA CGA GTA CCG–3′
		Reverse	5′–GTC ACT GGT CAA CTC CAG CAC–3′
*Adiponectin*	NM_009605.4	Forward	5′–GAT GGC ACT CCT GGA GAG AA–3′
		Reverse	5′–TCT CCA GGC TCT CCT TTC CT–3′
*HSL*	NM_001039507	Forward	5′–CAG AAG GCA CTA GGC GTG ATG–3′
		Reverse	5′–GGG CTT GCG TCC ACT TAG TTC–3′
*LPL*	NM_008509	Forward	5′–ATC GGA GAA CTG CTC ATG ATG A–3′
		Reverse	5′–CGG ATC CTC TCG ATG ACG AA–3′
*β-actin*	NM_007393.4	Forward	5′–CGT GCG TGA CAT CAA AGA GAA–3′
		Reverse	5′–GCT CGT TGC CAA TAG TGA TGA–3′

## Data Availability

Data are contained within the article.

## Supplementary Materials

The following are available online at https://www.mdpi.com/article/10.3390/ijms22179400/s1.
