# Supplementary figures and images for: Farrerol Induces Cancer Cell Death via ERK Activation in SKOV3 Cells and Attenuates TNF-α-Mediated Lipolysis
Source: Int J Mol Sci. 2021 Aug 30;22(17):9400. doi: 10.3390/ijms22179400 (PMC8430798; doi:10.3390/ijms22179400)

Figure S1

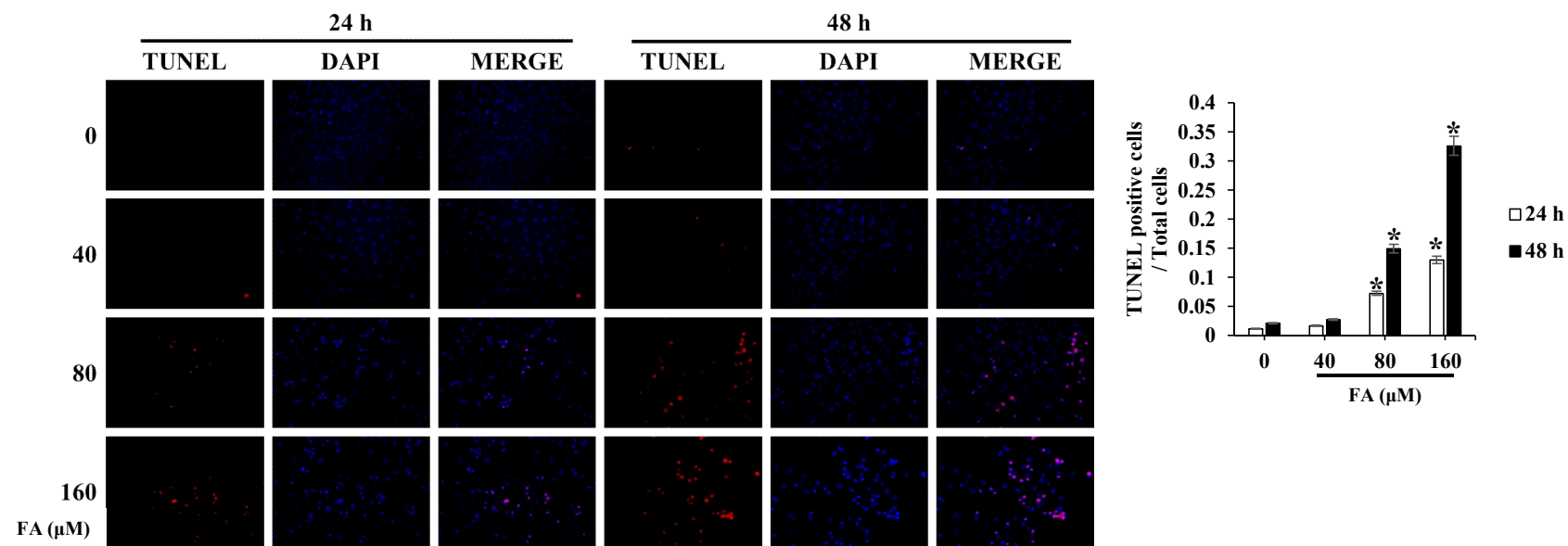

Figure S2

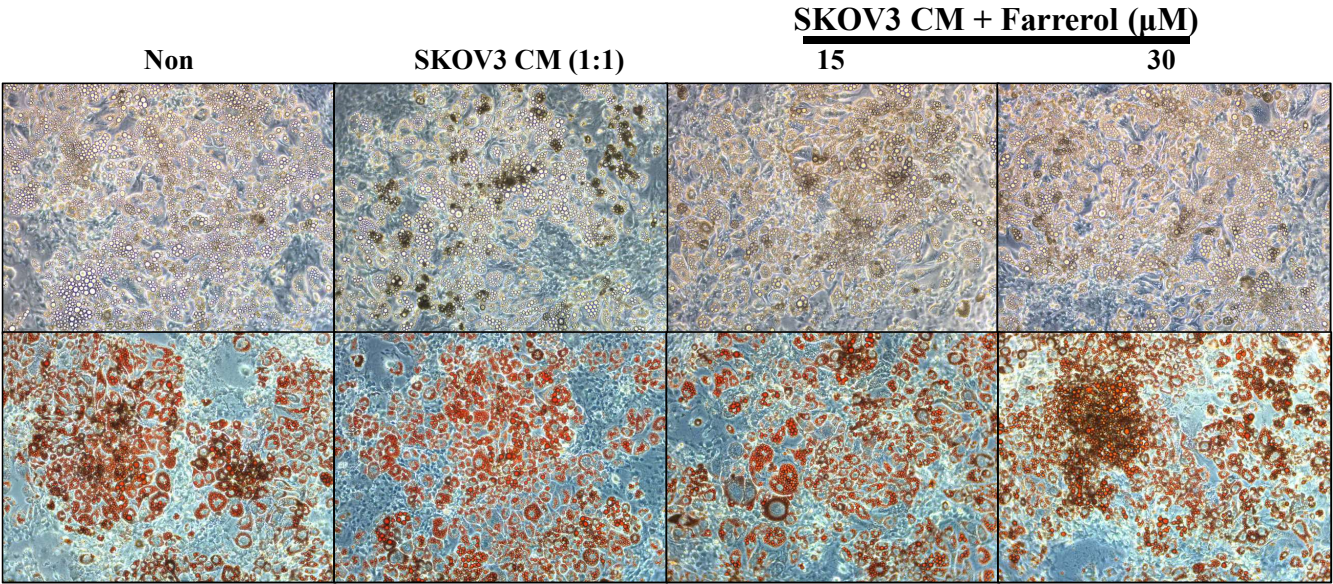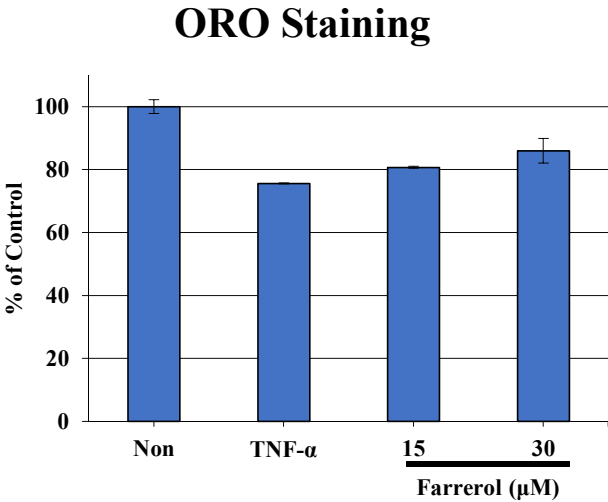

Supplement: Supplementary file 1 [file ijms-22-09400-s001.zip › ijms-1347626-supplementary.pdf]
